# Supplementary material for: The ubiquitin‐conjugating enzyme UBE2QL1 coordinates lysophagy in response to endolysosomal damage
Source: EMBO Rep. 2019 Aug 21;20(10):e48014. doi: 10.15252/embr.201948014 (PMC6776906; doi:10.15252/embr.201948014)
Supplement: Supplementary file 1 — Appendix [file EMBR-20-e48014-s001.pdf]

APPENDIX

The ubiquitin-conjugating enzyme UBE2QL1 coordinates lysophagy in response to endolysosomal damage

Lisa Koerver[1], Chrisovalantis Papadopoulos[1,5], Bin Liu[2,5], Bojana Kravic[1,5], Giulia Rota[1], Lukas Brecht[3], Tineke Veenendaal[4], Mira Polajnar[3], Anika Bluemke[1], Michael Ehrmann[1], Judith Klumperman[4], Marja Jäättelä[2], Christian Behrends[3,*], Hemmo Meyer[1,*]

[1] *Centre for Medical Biotechnology, Faculty of Biology, University of Duisburg-Essen, Germany*
[2] *Cell Death and Metabolism Unit; Center for Autophagy, Recycling and Disease; Danish Cancer Society Research Center; Copenhagen, Denmark*
[3] *SyNergy, Ludwig-Maximilians-Universität München, Feodor-Lynen Str. 17, 81377 München, Germany*
[4] *Section Cell Biology, Center for Molecular Medicine, University Medical Center Utrecht, Utrecht University, Utrecht, The Netherlands*
[5] *These authors contributed equally to this work.*

[*] *Corresponding authors:*
hemmo.meyer@uni-due.de, Christian.Behrends@mail03.med.uni-muenchen.de

Appendix Table of Contents:

Appendix Figure Legends	page 1
Appendix Figure S1	page 3
Appendix Figure S2	page 4
Appendix Figure S3	page 5
Appendix Figure S4	page 6

Appendix Fig S1 – Depletion efficiencies for all siRNAs targeting UBE2QL1. (related to figure 1)

A Western blot analysis of the depletion efficiency of all UBE2QL1 siRNAs used in the study. Cells were transfected with control siRNA (Ctrl), single UBE2QL1 siRNAs from the screen (#1-#4) or an additional siRNA targeting the open reading frame of UBE2QL1 (#5), and lysates were probed with an antibody to UBE2QL1 as indicated. GAPDH was probed as loading control.

Appendix Fig S2 – UBE2QL1 recruitment to lysosomes upon damage is independent of Gal3 and Gal8. (related to figure 4)

A HeLa cells were transfected with siRNAs targeting Gal3 or Gal8 individually, or both together for 48 h and either LLOMe or control-treated (untreated) for 3 h. Cells were fixed and immuno-stained for LAMP1 along with an antibody specific for UBE2QL1 as indicated and imaged by confocal laser scanning microscopy. Scale bar: 20 µm.

B Automated quantification of (A). Shown are the Pearson's correlation coefficients (P.C.C.) representing colocalization of endogenous UBE2QL1 and LAMP1. Graphs represent data from three independent experiments with ≥ 35 cells per condition (mean ± SD). N.S. (One-way-ANOVA with Bonferroni's multiple comparison test).

C Western blot analysis of depletion efficiency of Gal3 and Gal8 siRNAs. Cells were transfected with indicated siRNAs and lysates were probed with antibodies specific to Gal3 and Gal8 as indicated. GAPDH was probed as loading control.

Appendix Fig S3 – Basal lysosomal damage in UBE2QL1 or ATG5 and ATG7 depleted cells. (related to figure 6)

A Experiments as in Fig. 6A with longer treatment. HeLa cells were transfected with UBE2QL1 siRNA #4 or siCtrl for 72 h (rather than 60 h), fixed and stained using antibodies specific for Gal3 and LAMP1. Images were obtained by confocal laser scanning microscopy. Arrows indicate colocalizing vesicles. Scale bars: 10 µm, 2 µm for inlays.

B HeLa cells were transfected with siRNAs targeting ATG5, ATG7 or siCtrl for 72 h and stained and imaged as in A. Scale bars: 10 µm.

C Western blot analysis of depletion efficiencies of ATG5 and ATG7 siRNAs. Cells were transfected as in B but lysed and probed with antibodies for ATG5 and ATG7 (the asterisk indicates an unspecific band). GAPDH was probed as a loading control.

Appendix Fig S4 - Lysosomal damage caused by Terfenadine is exacerbated by UBE2QL1 depletion. (related to figure 6).

A HeLa cells stably expressing mCherry-Gal3 were transfected with indicated UBE2QL1 siRNAs for 60 h, treated with 8 µM Terfenadine for 24 h and analyzed by confocal microscopy. Scale bar: 10 µm.

A

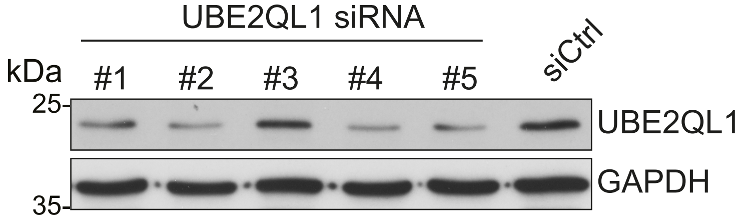

Appendix Figure S1

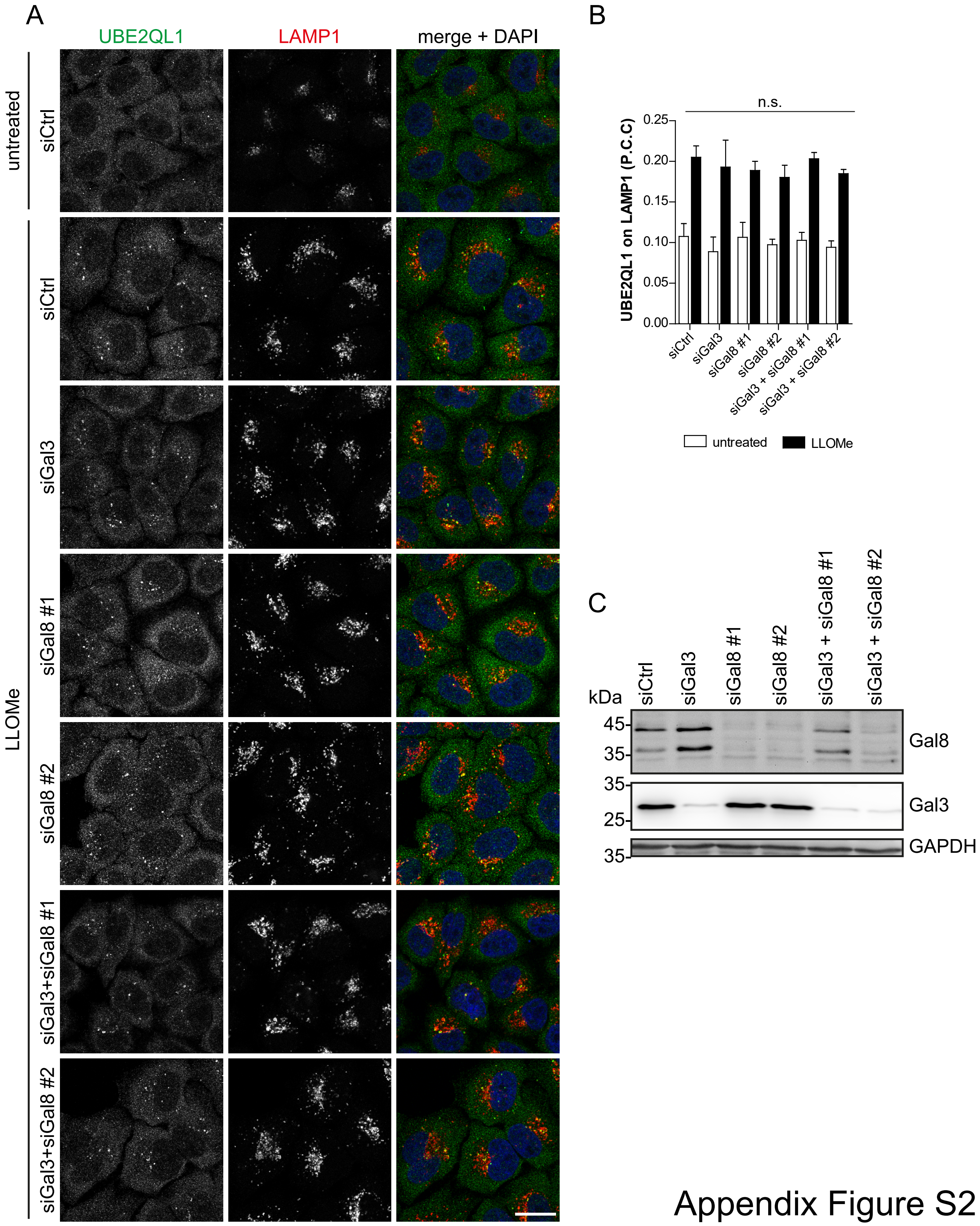

Appendix Figure S2

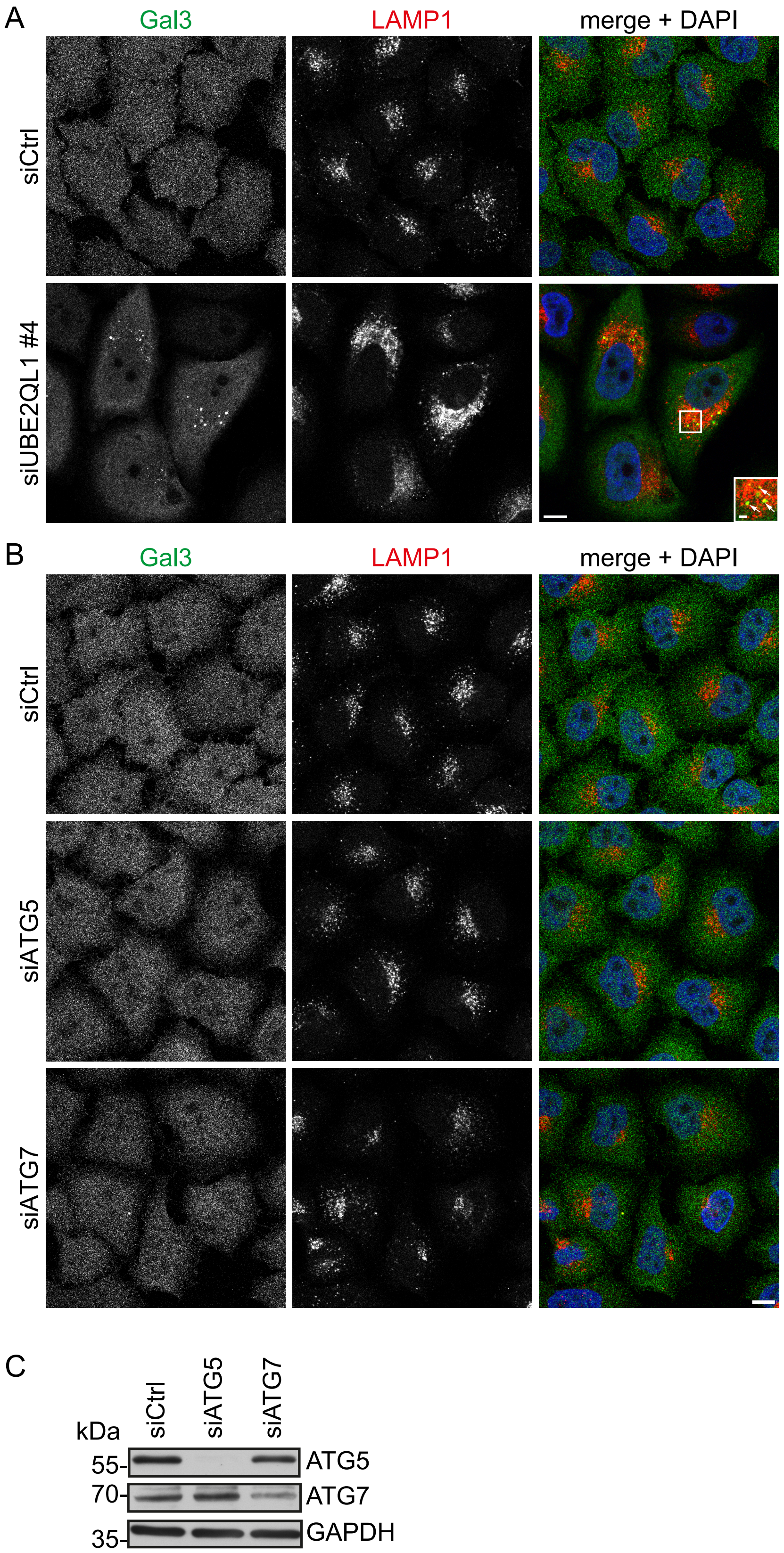

Appendix Figure S3

A

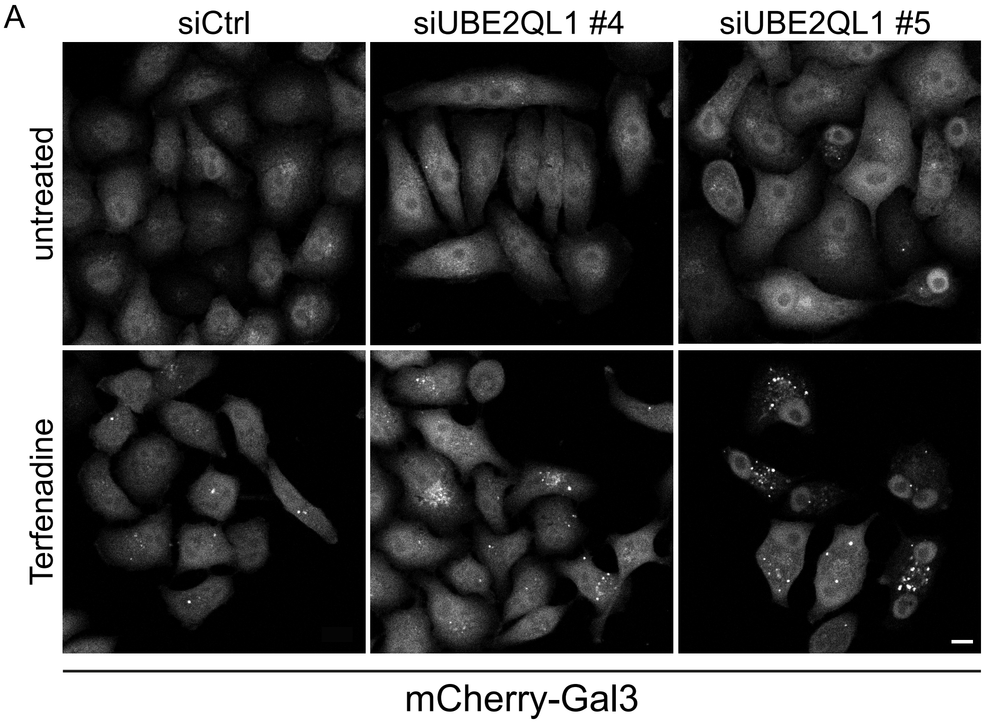

Appendix Figure S4
