# Supplementary material for: The ubiquitin‐conjugating enzyme UBE2QL1 coordinates lysophagy in response to endolysosomal damage
Source: EMBO Rep. 2019 Aug 21;20(10):e48014. doi: 10.15252/embr.201948014 (PMC6776906; doi:10.15252/embr.201948014)
Supplement: Supplementary file 2 — Expanded View Figures PDF [file EMBR-20-e48014-s002.pdf]

Expanded View Figures

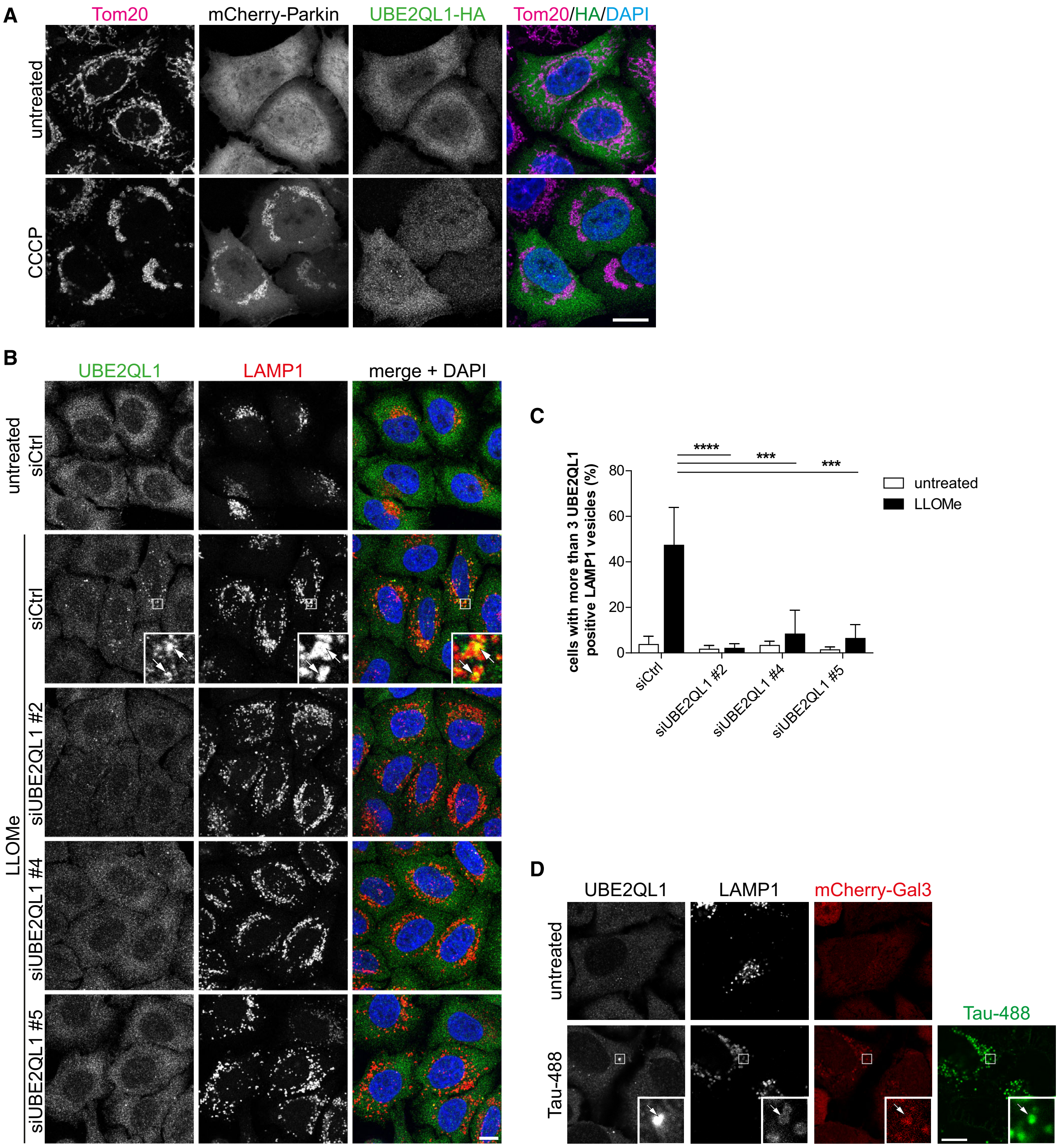

Figure EV1.

 © 2019 The Authors

Figure EV1. UBE2QL1 translocates specifically to damaged lysosomes (related to Fig 1).

A Immunofluorescence analysis of UBE2QL1-HA in cells with depolarized mitochondria. HeLa cells transiently transfected with mCherry-Parkin and UBE2QL1-HA were treated with 10 μM *Carbonyl cyanide 3-chlorophenylhydrazone* (CCCP) or DMSO alone (untreated) for 4 h, fixed and stained with antibodies specific for the mitochondrial protein Tom20 and HA. Note that there is no translocation of UBE2QL1-HA to depolarized mitochondria. Scale bar: 10 μm.

B HeLa cells that were transfected with control (Ctrl) or UBE2QL1-targeting siRNAs for 60 h and treated with LLOMe or EtOH alone (untreated) for 3 h were processed for immunofluorescence microscopy with antibodies against endogenous UBE2QL1 and LAMP1. Note that the UBE2QL1 signal colocalizing with LAMP1 in LLOMe-treated cells is suppressed by UBE2QL1 depletion indicating its specificity. Arrows indicate colocalizing vesicles. Scale bar: 10 μm.

C Automated quantification of (B). Percentage of cells with more than 3 UBE2QL1-positive LAMP1 vesicles. Graph represents data from three independent experiments with ≥ 50 cells per condition (mean ± SD). $^{***}P < 0.001$; $^{****}P < 0.0001$ (one-way ANOVA with Bonferroni's multiple comparison test).

D UBE2QL1 localizes to late endosomes/lysosomes damaged by endocytosed tau fibrillar tangles. HeLa cells stably expressing mCherry-Gal3 were incubated with Alexa488-labeled tau fibrils for 1 day. Cells were fixed and stained with indicated antibodies to UBE2QL1 and LAMP1 to detect endogenous UBE2QL1 on damaged lysosomes. Note colocalization of UBE2QL1 with tau-containing LAMP1 vesicles that are positive for Gal3 indicating damage. Arrow indicates colocalizing vesicle. Scale bar: 10 μm.

© 2019 The Authors

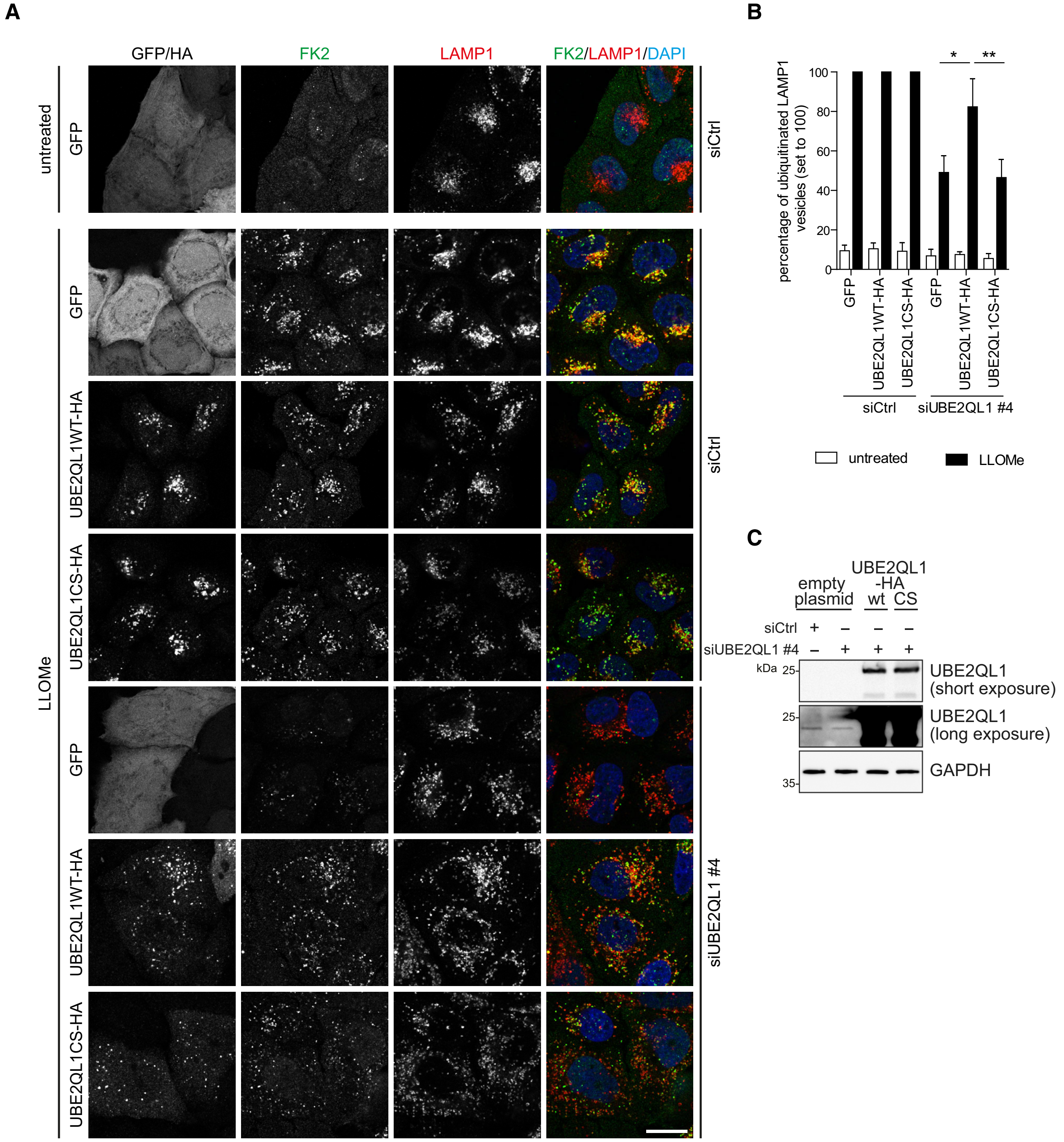

Figure EV2. Rescue of effects of UBE2QL1 depletion on lysosomal ubiquitination (related to Fig 2).

A Rescue of effects of UBE2QL1 depletion on lysosomal ubiquitination after damage. HeLa cells were transfected with siCtrl or siUBE2QL1 #4 for a total of 72 h. 24 h after beginning of siRNA treatments, cells were transfected with expression plasmids for UBE2QL1 wild-type (WT) or the catalytic inactive mutant C88S (CS) tagged with HA, or GFP alone as control as indicated. Lysosomal damage was induced by 250 μM LLOMe or EtOH alone (untreated) for 3 h. Cells were fixed and processed for immunofluorescence confocal laser microscopy with antibodies specific for the HA-tag, polyubiquitin (FK2), and LAMP1. Scale bar: 20 μm.

B Automated quantification of (A). FK2 and LAMP1 vesicles were identified, and the percentage of LAMP1 vesicles that were positive for ubiquitin was determined. The LLOMe-treated siCtrl control was set to 100% for each type of overexpressed protein. The graph represents data from three independent experiments with ≥ 25 cells per condition (mean ± SD). $^{*}P < 0.05$; $^{**}P < 0.01$ (one-way ANOVA with Dunnett's multiple comparison test).

C Western blot analysis with UBE2QL1 antibody of indicated samples related to the rescue experiment in (A). Note that the band of endogenous UBE2QL1 is only visible after long exposure. GAPDH was probed as a loading control.

 © 2019 The Authors

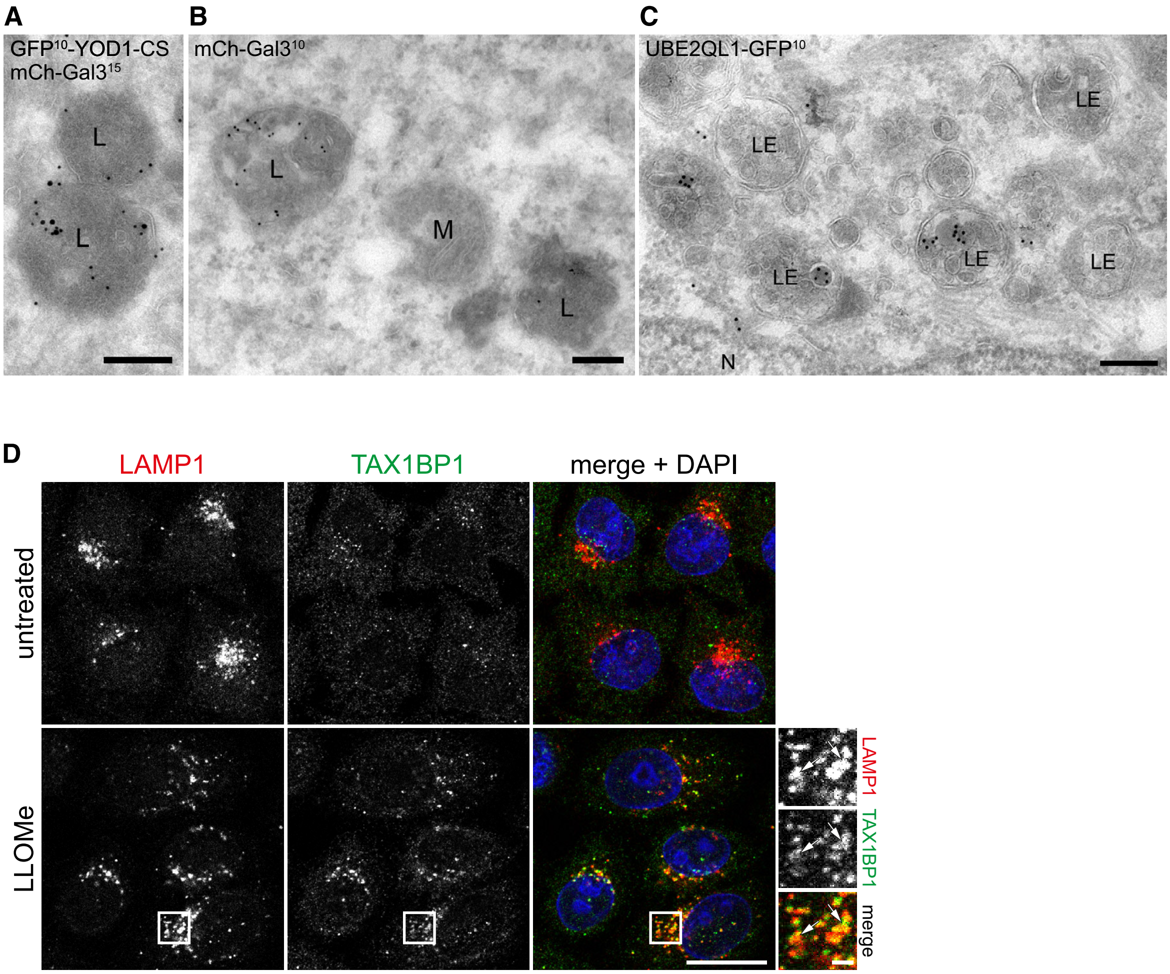

Figure EV3. Immuno-electron microscopy of damaged lysosomes and TAX1BP1 localization upon lysosomal damage (related to Fig 4).

A–C Immuno-electron microscopy of LLOMe-treated HeLa cells. Cells in (A) and (B) overexpress dominant-negative C160S mutant of GFP-YOD1 and mCherry-Gal3, and were immunostained for Gal3 and GFP. (A) GFP-YOD1 (10 nm gold particles) colocalizes with mCherry-Gal3 (15 nm gold particles) in the lumen of lysosomes (L). (B) mCherry-Gal3 (10 nm gold particles) positive lysosomes (at the left) are morphologically indistinguishable from galectin-negative lysosomes (right). (C) UBE2QL1-GFP (10 nm gold particles) in late endosome/lysosomal compartments (LE). N = nucleus, M = mitochondrion. Scale bars: 200 nm.

D HeLa cells were treated with LLOMe for 1 h. After methanol fixation, TAX1BP1 and LAMP1 were stained with specific antibodies and images were obtained by confocal microscopy. Note the recruitment of TAX1BP1 to lysosomes upon LLOMe treatment. Arrows indicate colocalizing vesicles. Scale bar: 20 μm and 2 μm for inlays.

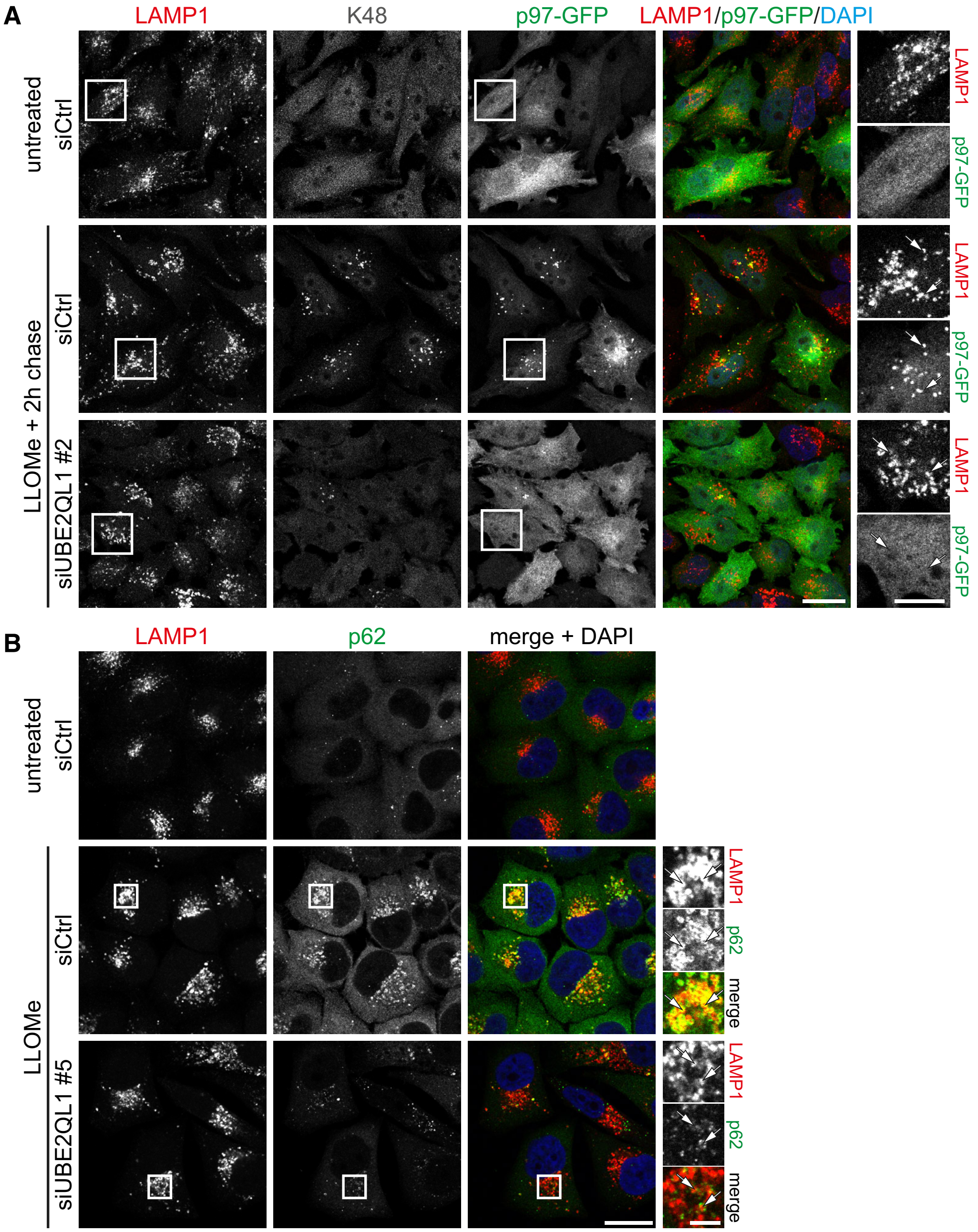

Figure EV4. UBE2QL1 abrogates recruitment of VCP/p97 and reduces accumulation of p62 (related to Fig 5).

A Immunofluorescence images for UBE2QL1 siRNA #2 corresponding to Fig 5A with the first two rows of micrographs (siCtrl) being identical. Scale bar: 20 μm.

B Immunofluorescence images for UBE2QL1 siRNA #5 corresponding to Fig 5C, with the second row of micrographs (siCtrl—LLOMe) being identical. Scale bar: 20 μm.

© 2019 The Authors

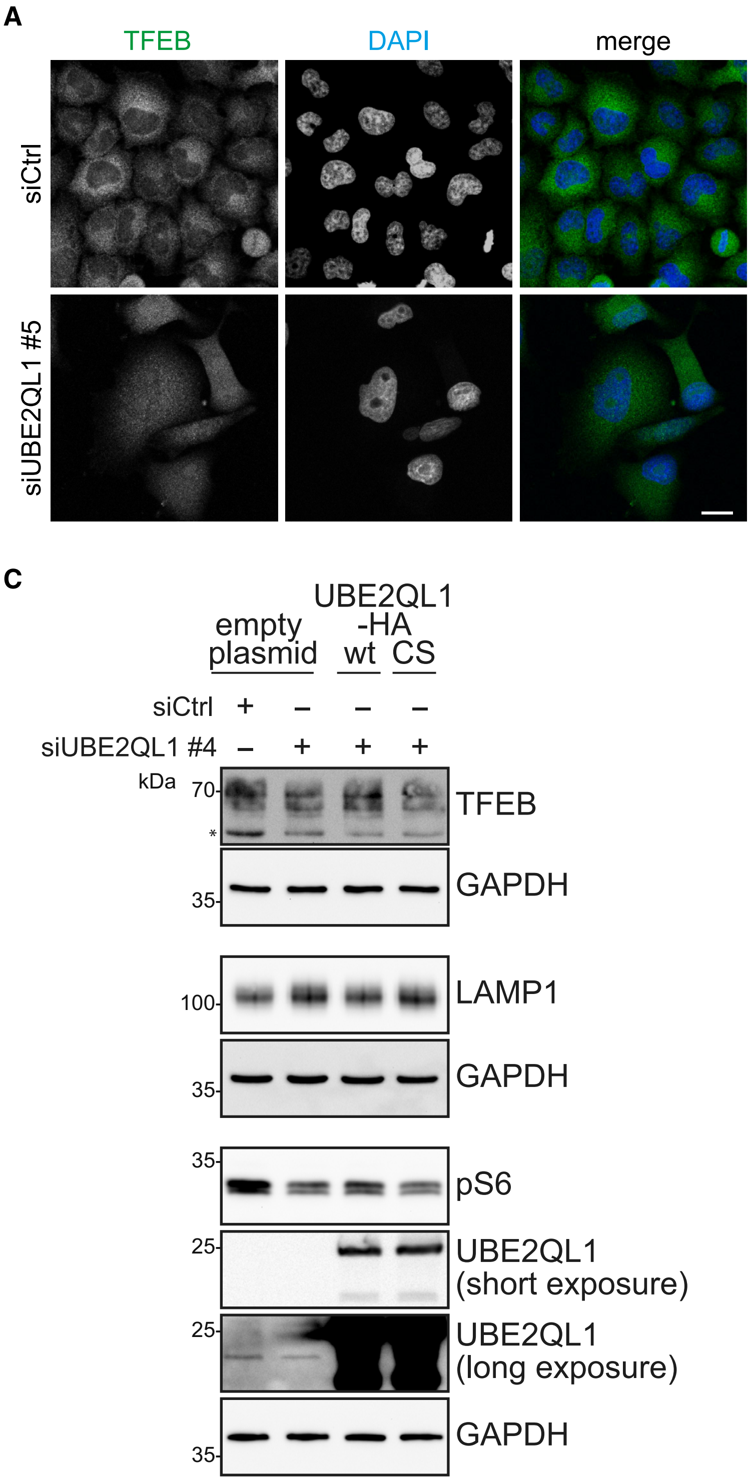

Figure EV5. UBE2QL1 is essential for maintenance of lysosomal integrity (related to Fig 7).

A Immunofluorescence images for UBE2QL1 siRNA #5 corresponding to Fig 7A, with the first row of control cells being identical. Scale bar: 20 μm.

B Immunofluorescence images for UBE2QL1 siRNA #5 corresponding to Fig 7C, with the first row being identical. Scale bar: 20 μm.

C Rescue of the effects of UBE2QL1 depletion on lysosomal homeostasis by UBE2QL1 overexpression. Lysates from rescue experiments in Fig EV2C were probed with antibodies against TFEB, LAMP1, and pS6 to monitor the effects of overexpression of UBE2QL1 WT and CS mutant in UBE2QL1-depleted cells. The blots for UBE2QL1 and GAPDH (identical to Fig EV2C) are shown for clarity. Lysates used for all blots are from the same experiment. The asterisk indicates an unspecific band.

Source data are available online for this figure.

© 2019 The Authors
